# Longitudinal Digital Phenotyping of Multiple Sclerosis Severity Using Passively Sensed Behaviors and Ecological Momentary Assessments: Real-World Evaluation

**DOI:** 10.2196/70871

**Published:** 2025-06-03

**Authors:** Zongqi Xia, Prerna Chikersal, Shruthi Venkatesh, Elizabeth Walker, Anind K Dey, Mayank Goel

**Affiliations:** 1 Department of Neurology University of Pittsburgh Pittsburgh, PA United States; 2 School of Computer Science Carnegie Mellon University Pittsburgh, PA United States; 3 Information School University of Washington Seattle, WA United States

**Keywords:** digital phenotyping, mobile sensing, wearable, multiple sclerosis, disability, depression, fatigue, sleep, machine learning, ecological momentary assessments, artificial intelligence

## Abstract

**Background:**

Longitudinal tracking of multiple sclerosis (MS) symptoms in an individual’s environment may improve self-monitoring and clinical management for people with MS. Conventional symptom tracking methods rely on self-reports and clinical visits, which can be infrequent, subjective, and burdensome. Digital phenotyping using passively collected sensor data from smartphones and fitness trackers offers a promising alternative for continuous, real-time symptom monitoring with minimal patient burden.

**Objective:**

We aimed to develop and evaluate a machine learning (ML)–based digital phenotyping approach to monitor the severity of clinically-relevant MS symptoms. We used passive sensing data to predict short-term fluctuations in patient-reported symptoms, including depressive symptoms, global MS symptom burden, severe fatigue, and poor sleep quality. Further, we examined the impact of incorporating behavioral context features and ecological momentary assessments on prediction performance.

**Methods:**

We conducted a 12- to 24-week longitudinal study involving 104 people with MS, collecting passive sensor and behavioral health data. Smartphone sensors recorded call activity, location, and screen use, while fitness trackers captured heart rate, sleep patterns, and step count. We extracted patient-level behavioral features and categorized them into 2 feature sets: one from the prediction period (called *action*) and one from the preceding period (called *context*). Using an ML pipeline based on support vector machines and AdaBoost, we evaluated the predictive performance of sensor-based models, both with and without ecological momentary assessment inputs.

**Results:**

Between November 16, 2019, and January 24, 2021, overall, 104 people with MS (women: n=88, 84.6%; non-Hispanic White: n=97, 93.3%; mean age 44, SD 11.8 years) from a clinic-based cohort completed 12 weeks of data collection, including a subset of 44 participants (women: n=39, 89%; non-Hispanic White: n=42, 95%; mean age 45.7, SD 11.2 years) who completed 24 weeks of data collection. In total, we collected approximately 12,500 days of passive sensor and behavioral health data from the participants. Among the best-performing models with the least sensor data requirement, the ML algorithm predicted depressive symptoms with an accuracy of 80.6% (*F*_1_-score=0.76), high global MS symptom burden with an accuracy of 77.3% (*F*_1_-score=0.78), severe fatigue with an accuracy of 73.8% (*F*_1_-score=0.74), and poor sleep quality with an accuracy of 72.0% (*F*_1_-score=0.70). Further, sensor data were largely sufficient for predicting symptom severity, while the prediction of depressive symptoms benefited from minimal active patient input in the form of responses to 2 brief questions on the day before the prediction point.

**Conclusions:**

Our digital phenotyping approach using passive sensors on smartphones and fitness trackers may help patients with real-world, continuous self-monitoring of common symptoms in their own environment and assist clinicians with better triage of patient needs for timely interventions in MS and potentially other chronic neurological disorders.

## Introduction

Multiple sclerosis (MS) is a leading cause of chronic neurological disability, affecting around 2.8 million people worldwide and >700,000 people in the United States, while causing high health and socioeconomic burdens [1-3]. People with MS may experience a variety of neurological symptoms involving the cognitive, motor, sensory, vision, bowel, or bladder domains, as well as symptoms of depression, fatigue, and sleep disturbance in their daily lives [4]. Comprehensive MS care involves timely symptom management, but clinicians’ awareness of symptoms often lags patient experience. Frequent symptom monitoring could improve clinical care and quality of life. However, active engagement with frequent longitudinal symptom monitoring is impractical for patients or clinicians. Given the pervasiveness of MS-related symptoms, symptom monitoring in the patient’s own environment coupled with effective prediction of symptom severity could facilitate triage for timely clinical intervention and reduce the delay in symptom management before worsening.

The digital phenotyping framework uses passively collected data from personal digital devices (eg, smartphones and fitness trackers) to quantify human behavior moment-by-moment in situ and predict individual health outcomes [5]. Previous works using passively sensed smartphone and wearable data to predict MS outcomes explored the feasibility of passive data collection and the preliminary association between sensed behaviors and standard rater-assessed clinical outcomes [6-14]. However, little is known regarding the clinical applicability of continuous longitudinal digital phenotyping to predict the severity of clinically relevant *patient-reported symptoms* in people with MS. Here, we proposed a machine learning (ML) approach that harnesses continuously and passively collected data from patients’ digital devices to predict short-term future symptoms. Specifically, we prioritized common MS neurological symptoms as well as symptoms of depression, fatigue, and sleep disturbance that collectively worsen the quality of life.

In this study, we used the concepts of *action features* and *context features* to better capture more recent versus less recent behaviors to be modeled. Action features represent a patient’s behaviors during the period immediately preceding a symptom assessment (eg, the preceding 2 or 4 weeks), while context features capture behaviors from an earlier period, providing a *historical* context for interpreting or contextualizing the patient’s more recent behavioral patterns. We also asked patients to complete brief self-reports of their instantaneous symptoms and experiences multiple times a day, to which we refer as ecological momentary assessments (EMAs) that provide additional snapshots of real-time symptoms to complement the passively collected sensor data.

The primary study goal was to test the feasibility of low-cost, continuous, and longitudinal symptom tracking in a patient’s own environment with minimal active patient engagement. Secondarily, we examined whether ML model performance based on passively collected sensor data would improve when (1) using behavioral features from the previous period (context features) to help the models contextualize the patient’s current behaviors in addition to behavioral features from the current period (action features), and (2) incorporating minimal active patient input via EMAs. These aspects of the study design in digital phenotyping of clinically relevant patient-reported symptoms differentiate from prior studies. Our approach may also inform the real-world application of long-term, continuous symptom tracking and real-world clinical prediction in chronic neurological conditions beyond MS. Integrating this digital health approach into routine clinical practice could enable more individualized disease monitoring, support clinical decision-making through real-time, data-driven insights, and improve quality of life. Using MS as an illustration of the potential clinical application of digital phenotyping in a chronic neurological disorder with multifaceted symptomatic manifestations, this study provides proof-of-concept that the broader adoption of wearable and smartphone-based monitoring systems in routine clinical practice could enhance symptomatic management in other complex chronic neurological disorders.

## Methods

### Participants and Study Period

The study included adults aged ≥18 years with a neurologist-confirmed MS diagnosis who owned a smartphone (Android or iOS) and enrolled in the Prospective Investigation of Multiple Sclerosis in the Three Rivers Region study, a clinic-based MS natural history cohort at the University of Pittsburgh [15-22].

Between November 16, 2019, and January 24, 2021, a total of 104 participants completed the data collection for a predefined period of 12 weeks, while 44 (42.3%) participants extended data collection for an additional 12 weeks to complete 24 weeks of data collection. None of the participants experienced acute relapses during the study period.

### Ethical Considerations

The institutional review boards of the University of Pittsburgh (STUDY19080007) and Carnegie Mellon University (STUDY2019-00000037) approved the study. All participants provided written informed consent. To protect confidentiality, we removed identifiable information (eg, names and contact information) from sensor and questionnaire data before analysis.

### Overview of the Digital Phenotyping Approach

To briefly summarize the overall approach, we used passively and continuously collected data from participants’ own digital devices, including 3 *smartphone sensors* (calls, locations, and screen use) and 3 *fitness tracker sensors* (heart rate, sleep, and steps), to predict short-term future patient-reported symptoms of MS-related global neurological symptom burden, depression, fatigue, and sleep quality. To assess the added predictive utility of EMAs, which were brief surveys for “repeated sampling of participants’ current behaviors and experiences in real time in participants’ natural environments” [23,24], we administered EMAs 3 times per day through a mobile app asking 2 5-point Likert scale questions that took <15 seconds on average to respond. To capture the real-world fluctuation in symptom severity, we divided each participant’s collected data into discrete consecutive periods (eg, 2 or 4 weeks) for rolling predictions of patient-reported symptoms. We used participants’ responses to validated symptom questionnaires during the same period as the ground truth of symptom severity. We computed features from the sensor and EMA data and classified features as action versus context based on the temporal relationship between features and patient-reported symptom severity at each period. *Action features* captured a person’s activity and behaviors during the period immediately preceding the *next* point of symptom severity prediction. *Context features* captured a person’s activity and behaviors during the period immediately preceding the *previous* prediction point, that is, the context of a participant’s action features. We then used (1) action features or (2) action and context features to predict symptom severity.

### Sensor and EMA Data Collection

At enrollment, the study team helped each participant install a custom-built mobile app on their smartphone. In parallel, the study team provided each participant with a Fitbit Inspire HR device to wear. Participants kept the Fitbit after study completion. We asked participants to always carry their smartphones, wear fitness trackers, and keep their devices charged.

The mobile app was based on the AWARE framework [25], and provided the backend and network infrastructure for unobtrusively collecting call logs (eg, incoming, outgoing, and missed calls), locations, and screen use (ie, when the screen status changed to on or off and locked or unlocked) of the smartphone sensors. The fitness tracker sensors captured heart rate, sleep status (eg, asleep, awake, restless, or unknown), and the number of steps. Data from AWARE were deidentified and automatically transferred over Wi-Fi to a study server at regular intervals. Data from the Fitbit were retrieved using the Fitbit application programming interface at the end of each participant’s data collection.

Calls and screen use were event-based sensor streams, whereas location, heart rate, sleep, and steps were time series sensor streams. We sampled location coordinates at 1 sample per 10 minutes and heart rate, sleep, and steps at 1 sample per minute.

Throughout the study duration, the mobile app alerted and directed participants 3 times a day to complete a brief EMA survey within the app. EMA surveys took <15 seconds to complete on average. The 2 recurring questions were as follows: (1) “How depressed do you feel?” and (2) “How tired do you feel?” Participants responded to each EMA question using a Likert scale from 0 to 4, with 0 indicating the least and 4 indicating the most depressed or tired feeling. The EMA responses were transmitted to the study server.

### Questionnaire Deployment for Assessing Symptom Severity

#### Overview

Participants completed web-based questionnaires using the secure Research Electronic Data Capture system [26,27]. To assess the severity of clinically relevant symptoms, we used standardized patient-reported outcome questionnaires validated in people with MS. To harmonize the periods across participants, all participants completed a baseline questionnaire assessing demographics and clinical profiles on the Saturday following enrollment. Beyond the baseline, participants completed additional questionnaires at regular intervals (eg, every 2 or 4 weeks from the first Saturday) as appropriate for assessing each standard patient-reported symptom type throughout the data collection period.

#### Depressive Symptoms

To measure the severity of depression symptoms, participants completed the Patient Health Questionnaire-9 (PHQ-9) once every 2 weeks [28]. The PHQ-9 asked for symptoms in the preceding 2 weeks, whereas the other questionnaires in this study asked for symptoms in the preceding 4 weeks. PHQ-9 scores ranged from 0 to 3, with higher scores indicating more severe depressive symptoms.

#### Global MS Neurological Symptom Burden

To measure the severity of the global MS-related neurological symptom burden, participants completed the Multiple Sclerosis Rating Scale-Revised (MSRS-R) once every 4 weeks [29]. MSRS-R assessed 8 neurological domains (ie, walking, upper limb function, vision, speech, swallowing, cognition, sensory, bladder, and bowel function). Each domain could score from 0 to 4, with 0 indicating the absence of symptoms and 4 indicating the greatest symptom severity. The total score (0-32) indicates the global MS-related neurological symptom burden.

#### Fatigue Impact

To measure the severity of fatigue, participants completed the Modified Fatigue Impact Scale-5 (MFIS-5) once every 4 weeks [30]. MFIS-5 assessed the impact of fatigue on cognitive, physical, and psychosocial function. Each item in MFIS-5 could score from 0 (never) to 4 (almost always) on a 5-point Likert scale, with higher scores indicating more severe fatigue.

#### Sleep Quality

To measure the severity of sleep disturbances, participants completed the Pittsburgh Sleep Quality Index (PSQI) once every 4 weeks [31]. The 19 items of PSQI generated 7 component scores (each on a 0-3 scale) and one composite score (0-21), with higher scores indicating poorer sleep quality.

Binary indicators of symptom severity likely have more practical real-world clinical utility in assisting patient self-monitoring and facilitating clinician triage for symptom intervention. For each symptom type, we dichotomized the score to the respective standardized questionnaire using specific thresholds to classify symptom severity. For global MS neurological symptom burden, we dichotomized MSRS-R scores as ≥6.4 (higher burden) versus <6.4 (lower burden). For depressive symptoms, we dichotomized PHQ-9 scores as ≥5 (presence of depressive symptoms) versus <5 (absence of depressive symptoms). For fatigue, we dichotomized MSIF-5 scores as ≥8 (greater fatigue) versus <8 (lower fatigue). For sleep quality, we dichotomized PSQI scores as ≥9 (poorer sleep quality) and <9 (better sleep quality). For depressive symptoms and sleep quality, the binary thresholds were based on previous consensus [28,32]. For global MS neurological symptom burden and fatigue, we calculated the respective median scores in the entire dataset. We used the median scores as the thresholds, given the lack of consensus from the literature. Thus, throughout the data collection, each participant had a consecutive series of binary symptom severity status (ie, every 2 weeks for depressive symptoms and every 4 weeks for global MS symptom burden, fatigue, and sleep quality).

### ML Modeling

#### Overview

**Figure 1:**
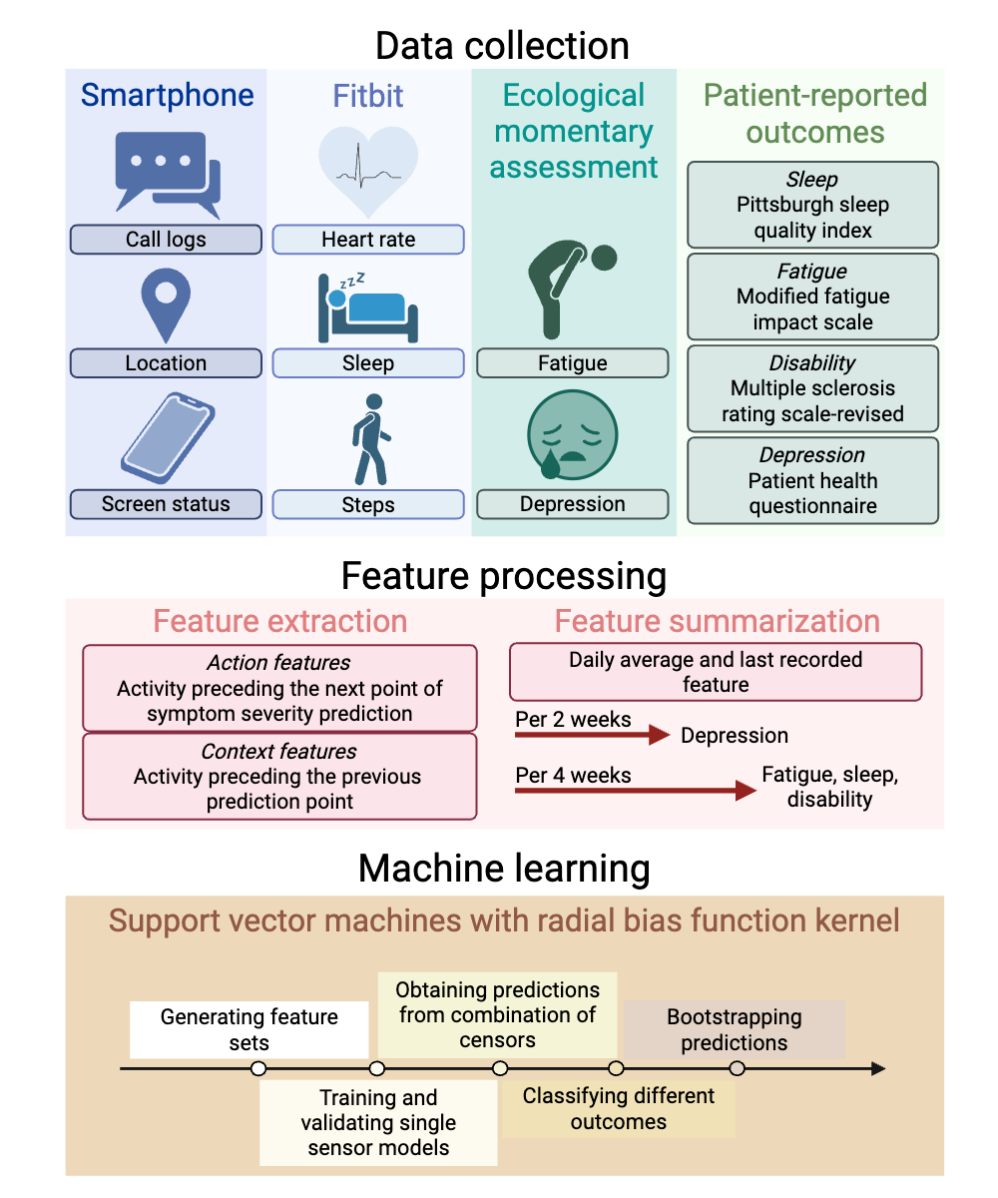
Data processing and analysis pipeline. The pipeline for predicting depressive symptoms (Patient Health Questionnaire-9) every 2 weeks, and global multiple sclerosis neurological symptom burden (Multiple Sclerosis Rating Scale-Revised), fatigue (Modified Fatigue Impact Scale-5), and sleep quality (Pittsburgh Sleep Quality Index) every 4 weeks, used passively collected sensor data from smartphones and fitness trackers as well as ecological momentary assessments (EMAs). We ran the pipeline for 2 types of EMA features (average and presurvey EMAs) and 2 types of feature matrices (action and action and context). For each sensor, every feature was extracted from 15 temporal slices over 2- or 4-week periods. First, raw data from the device sensor were preprocessed and filtered by time-of-the-day and days-of-the-week. Features were then extracted from the selected raw data. For EMA, we used a similar approach (as for processing sensor data) to calculate the average EMA and presurvey EMA. Action features were features from the period immediately preceding the prediction point, whereas context features were from the period preceding the “action period”.

Briefly, the data processing and analysis pipeline required the following steps (Figure 1). First, we extracted features from sensor and EMA data to generate action and context features. Second, we improved data quality by handling missing features. Finally, we implemented an ML pipeline to predict the severity of each patient-reported symptom on a rolling basis (ie, every 2 weeks for depressive symptoms and every 4 weeks for global MS neurological symptom burden, fatigue, and sleep quality) using action features or action and context features in the following iterations: (1) 1-sensor models, each containing features from one out of the 6 sensor types; (2) the best combination of the 1-sensor models; (3) the best combination of 1-sensor models plus EMA.

#### Feature Extraction and Engineering

##### Overview

From the smartphone and fitness tracker sensors, we computed 6 types of features from different sensors (ie, calls, heart rate, location, screen use, sleep, and steps), given their known potential to inform behaviors relevant to symptoms of depression [33-38], fatigue [10], poor sleep quality [39,40], and crucial MS neurological symptoms, such as decreased mobility [13]. The “calls” feature captured communication patterns. The “heart rate” and “steps” features captured the extent of physical activity. The “location” feature captured mobility patterns. The “screen use” feature potentially captured the ability for concentration [41,42] and the extent of sedentary behavior [43] with caveats for people with MS and people with other chronic neurological disorders who may experience impairment with upper limb or fine motor functions. The “sleep” feature captured sleep duration and patterns, from which we could infer sleep disturbance (eg, insomnia or hypersomnia) [44]. Multimedia Appendix 1 (section A.1) provides details of sensor feature extraction and engineering. For sensor features over time periods (eg, every 2-week or 4-week period; Figure 1), we calculated the daily average value of each sensor feature. Given the diversity of behaviors with ephemeral and sustained changes in people with MS, it is crucial to initialize the model with a large feature set. While these features captured individual or overlapping behaviors, the feature selection stage of our ML pipeline removed redundant features.

For EMA responses during the same time periods (eg, every 2-week or 4-week period; Figure 1), we obtained 2 types of EMA features. The “average EMA” was the daily average value of each EMA question response during a given period. The “presurvey EMA” represented the value of the last response to each EMA question on the day before the administration of the questionnaire for assessing the patient-reported symptom during each period.

##### Temporal Slicing

The temporal slicing approach extracted sensor features from different time segments (Figure 1). From previous research, temporal slicing better defined the relationship between a sensor feature and depression severity [45,46]. Here, we collected all available data during each specific epoch or time segment of the day (all day; night, midnight-6 AM; morning, 6 AM-noon; afternoon, noon-6 PM; and evening, 6 PM-midnight) and on specific days of the week (all days of the week, weekdays only [Monday-Friday], and weekends only [Saturday-Sunday]) to achieve 15 data streams or *temporal slices.* For sensor or EMA features in each of the 15 temporal slices, we first computed daily features (of the temporal slice) and averaged daily features over either 2- or 4-week periods for prediction (ie, every 2 weeks to predict depressive symptoms and every 4 weeks to predict global MS neurological symptom burden, fatigue, and sleep quality). We concatenated the features from 15 temporal slices to derive the final feature matrix. We selected these slicing intervals based on circadian rhythms and established practice in passive sensing studies. This approach remains a practical and lightweight method to uncover possible time-of-day or day-of-week patterns in behavior that might otherwise be obscured by averaging over entire weeks or months.

##### Feature Matrix

After feature extraction, we created a feature matrix for each of the 6 sensors (calls, locations, screen use, heart rate, sleep, and steps) and each of the 2 EMA types (average and presurvey EMA), containing features for the 15 temporal slices in consecutive 2- or 4-week periods during each participant’s study follow-up. The “action” feature matrix captured each participant’s actions during the *current* (2- or 4-week) period. At the end of this period, we predicted the patient-reported symptom severity as the outcome. For each participant, we concatenated features from the *previous* (2- or 4-week) period, which captured the context for the current actions, with the “action” feature matrix to obtain the “action and context” feature matrix. Thus, to predict the outcome at the end of the i^th^ period at time T=iP where P=2 weeks or 4 weeks, the action feature matrix comprised features from time (i-1)P and time iP, whereas the action and context feature matrix comprised features from time (i-2)P and time iP (Figure 1).

#### Handling Missing Data

Missing sensor data could occasionally occur due to several reasons. Multimedia Appendix 1 (section A.2) describes the detailed approach for handling missing data.

#### ML Pipeline Using Action and Context Behavioral Features

##### Overview

We built ML models using support vector machines (SVMs) with radial basis function (RBF) kernels and validated our models using *leave-5-participants-out cross-validation* to mitigate overfitting. As an overview, the pipeline involved 6 steps. First, in the *generating feature sets* step, we created model configurations that enabled assessment of the utility of EMA features and contextual feature information. Second, we performed *training and validating 1-sensor and EMA-only models* step for each of the 6 sensor feature types (calls, heart rate, location, screen use, sleep, and steps) and either EMA feature type (average or presurvey EMA features). Third, during the *obtaining predictions from combinations of sensors* step, we combined detection probabilities from 1-sensor models to identify the best-performing combined sensor model. Fourth, during *obtaining predictions from combinations of sensors and EMA* step, we combined detection probabilities from 1-sensor models and an EMA-only model to identify the best-performing final model. Fifth, we performed the *classifying different outcomes* step by running the pipeline for each outcome. Finally, we performed a comparison of ML models using *bootstrapping predictions.*

##### Generating Feature Sets

We generated features for the different model configurations to assess the utility of EMA features and contextual feature information. For *EMA*, we used (1) no EMA information, (2) only presurvey EMA, or (3) average EMA values. For *context*, we either used (1) only action or (2) action and context. In total, there were 6 configurations based on these features.

##### Training and Validating 1-Sensor and EMA-Only Models

**Figure 2:**
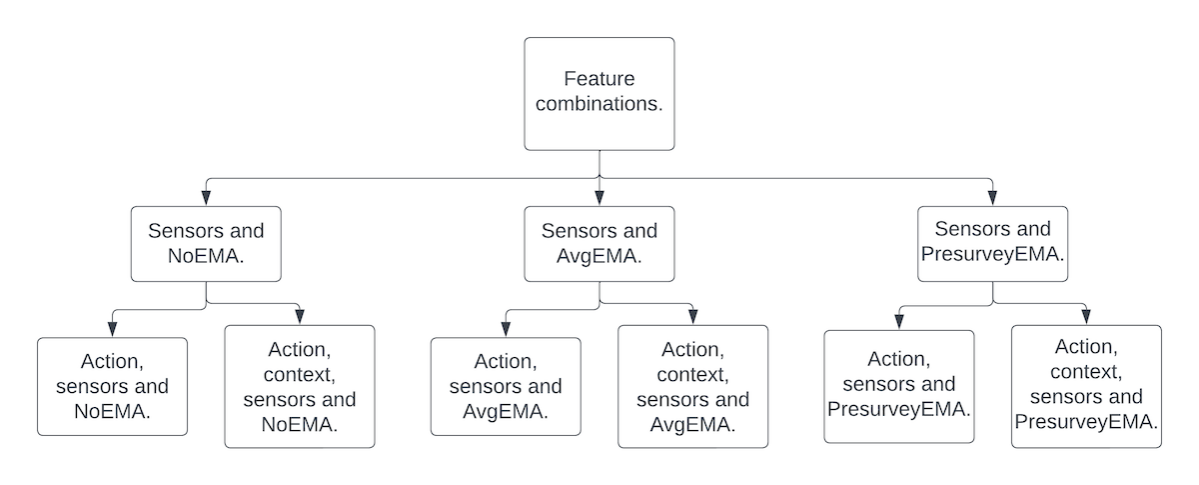
Feature combinations from sensors and ecological momentary assessments (EMAs).

For each sensor and EMA feature matrix, we built a model of the selected features from the given sensor or EMA type to predict an outcome (Figure 2). We trained models using an SVM classifier with RBF Kernel (SVM-RBF). We used leave-5-participants-out cross-validation to choose the regularization parameter for SVM-RBF. The folds were split in a stratified manner, and classes were balanced in the SVM-RBF to ensure that positive and negative classes of the binary outcomes were adequately represented. We chose the model with the best *F*_1_-score for a given outcome, which provided the prediction probabilities for the outcome. The process for one outcome was independent of the other outcomes.

##### Obtaining Predictions From Combinations of Sensors

We concatenated prediction probabilities from all six 1-sensor models into a single feature vector and entered it as input into an ensemble classifier, that is, AdaBoost with Decision Tree Classifier as a base estimator, which generated the final prediction for each outcome. For all outcomes, only the prediction probabilities of the positive label “1” were concatenated. The positive labels were the “presence of depressive symptoms” for depression, “high burden” for global MS neurological symptom burden, “severe fatigue” for fatigue, and “poor sleep quality” for sleep quality. We tuned the “n_estimators” (ie, the maximum number of estimators at which boosting was terminated) parameter during leave-5-participants-out cross-validation to achieve the best-performing combined model.

To analyze the contribution of each sensor combination, we implemented a feature ablation analysis by generating detection results for all possible combinations of 1-sensor models. For six 1-sensor models, there were 57 combinations of feature sets, as the total combinations = combinations with 2 sensors +...+ combinations with 6 sensors:



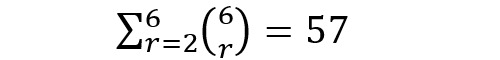



##### Obtaining Predictions From Combinations of Sensors and EMA-Only Models

We concatenated prediction probabilities from all six 1-sensor models and one EMA-only model into a single feature vector and entered it as input into an ensemble classifier using the same method for sensors (as described in aforementioned section) to train this combined classifier.

To analyze the utility of each sensor and EMA combination, we implemented a feature ablation analysis by generating detection results for all possible combinations of 1-sensor models and the EMA model. For six 1-sensor models and one EMA model, there were 120 combinations of feature sets, as the total combinations = combinations with 2 sensors or 1 sensor and EMA +...+ combinations with 6 sensors or EMA: 



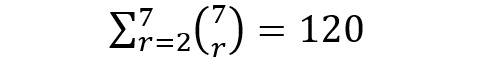



##### Classifying Different Outcomes

We ran the following pipeline independently for each of the 4 patient-reported symptoms as the outcomes, first using action-only features and then using action and context features: (1) training and validating six 1-sensor models without EMA and 57 combined models, (2) training and validating six 1-sensor models plus average EMA and 120 combined models, and (3) training and validating six 1-sensor models plus presurvey EMA and 120 combined models.

Each patient had multiple “samples” (ie, prediction periods) over the study duration. For each patient-reported symptom, we trained 6 final models based on whether the model included action versus action and context features or whether the model contained no EMA, average EMA, or presurvey EMA. Here, the “positive” label refers to the outcome of interest (eg, presence of depressive symptoms, presence of high global MS neurological symptom burden, presence of severe fatigue, or presence of poor sleep quality). For each final model of a given outcome, we reported the model performance of the best combination of sensors and EMA. We also reported the performance of baseline models (ie, a simple majority classifier whereby every point was assigned to whichever was in the majority in the training set) as well as models containing all 6 sensors or all 6 sensors plus 1 EMA type.

##### Comparing ML Models by Bootstrapping Predictions

For model performance metrics, we assessed accuracy and *F*_1_-score. *Accuracy* is the percentage of samples for which the model correctly predicted the outcome label. *F*_1_-score measures the harmonic mean of precision and recall. Precision is the positive predictive value, that is, the number of true positive labels divided by the number of all positive labels (true positive + false positive). Recall is sensitivity, that is, the number of true positive labels divided by the number of all samples that should have the positive labels (true positive + false negative). For each patient-reported symptom, we compared the bootstrapped accuracy and *F*_1_ scores among the 6 final models in a pairwise manner (30 comparisons). Specifically, we computed the 95% CIs of differences in their bootstrapped accuracy and *F*_1_-score. We performed hierarchical bootstrapping by randomly sampling (participant ID and prediction week) with replacement over 10,000 iterations. In each iteration, we took samples with the same (participant ID and prediction week) across the 2 models being compared and computed the difference in accuracy and difference in *F*_1_-score, respectively. After computing all iterations, we generated the 95% CIs of the difference in accuracy and the difference in *F*_1_-score (2-tailed alpha=.05). If one of the models in a pair was not statistically better than the other, we considered the model requiring the least amount of sensor and EMA data to be “better.”

## Results

### Patient Profile

The study included 104 people with MS who completed at least 12 weeks of data collection between November 2019 and January 2021. The subset of the participants who completed 24 weeks of data collection shared similar characteristics as the study cohort, which was largely representative of the larger clinic-based MS population (Table 1).

**Table 1:** Patient characteristics.

Characteristics		12 weeks (n=104)	24 weeks (n=44)	*P* value	
Age (y), mean (SD)		44 (11.8)	45.7 (11.2)	.42	
**Sex, n (%)**					.61
	Female	88 (84.6)	39 (88.6)		
	Male	16 (17.3)	5 (11.4)		
**Race, n (%)**					.97
	Asian	0 (0)	0 (0)		
	Black or African American	7 (6.7)	2 (4.5)		
	White	97 (93.3)	42 (95.5)		
	Not reported	0 (0)	0 (0)		
**Ethnicity, n (%)**					.99
	Non-Hispanic	104 (100)	44 (100)		
	Hispanic	0 (0)	0 (0)		
	Not reported	0 (0)	0 (0)		
Disease duration (y), mean (SD)		13.7 (10.1)	15.0 (10.5)	.48	
**Disease subtype, n (%)**					.32
	RRMS^a^ and precursors (RIS^b^ and CIS^c^)	100 (96.2)	44 (100)		
	PMS^d^	4 (3.8)	0 (0)		
**DMT^e^ efficacy, n (%)**					.39
	No DMT	27 (26.0)	11 (25.0)		
	Standard Efficacy	19 (18.3)	10 (22.7)		
	Higher Efficacy	58 (55.8)	23 (52.3)		
PHQ^f^-2 score, mean (SD)		0.79 (0.9)	0.77 (0.91)	.90	
PHQ-9 score^g^, mean (SD)		10.8 (4.2)	11.2 (4.3)	.60	
MSRS-R^h^ score, mean (SD)		7.4 (5.4)	7.9 (5.5)	.61	
MFIS^i^ score, mean (SD)		8.5 (4.7)	8.6 (4.6)	.91	
PSQI^j^ score, mean (SD)		9.8 (4.0)	10.2 (4.0)	.58	

^a^RRMS: relapsing-remitting multiple sclerosis.

^b^RIS: radiologically isolated syndrome.

^c^CIS: clinically isolated syndrome.

^d^PMS: progressive multiple sclerosis.

^e^DMT: disease-modifying therapies.

^f^PHQ: Patient Health Questionnaire.

^g^PHQ-9 was only deployed when the participants scored ≥1 on the PHQ-2.

MSRS: Multiple Sclerosis Rating Scale-Revised.

^i^MFIS: Modified Fatigue Impact Scale-5.

^j^PSQI: Pittsburgh Sleep Quality Index.

### Predicting Outcomes Using Action and Context Features From Sensor and EMAs

#### Overview

We reported the accuracy and *F*_1_-score of the ML pipeline for predicting each type of patient-reported symptom using the best-performing sensor and EMA combinations (ie, the set of sensors and average or presurvey EMA) for models trained on action-only features and action and context features (Table 2). Separately, we reported the performance of *individual* 1-sensor, average EMA, and presurvey EMA models (Table S1 in Multimedia Appendix 1) as well as models combining all 6 sensors, 6 sensors and average EMA, or 6 sensors and presurvey EMA (Table S2 in Multimedia Appendix 1). Finally, we indicated the best combination of sensors and EMA selected for each model type (Table S3 in Multimedia Appendix 1).

**Table 2:** Performance of the machine learning pipeline^a^.

Model	Depression			MS^b^ symptom burden			Fatigue			Sleep quality	
	Accuracy (%)	*F*_1_-score	Accuracy (%)		*F*_1_-score	Accuracy (%)		*F*_1_-score	Accuracy (%)		*F*_1_-score
Action-Only and NoEMA^c^	75	0.68	77		0.77	68		0.68	72		0.7
Action and Context and NoEMA	75	0.69	74		0.74	74		0.74	69		0.67
Action-Only and AvgEMA	81	0.77	78		0.78	72		0.73	74		0.71
Action and Context and AvgEMA	81	0.77	80		0.8	76		0.76	73		0.69
Action-Only and PresurveyEMA	81	0.76	78		0.78	68		0.68	72		0.7
Action and Context and PresurveyEMA	81	0.77	75		0.75	77		0.78	74		0.7

^a^We used the best sensor or sensor and EMA combinations for predicting the 4 patient-reported symptoms in people with MS: depressive symptom, global MS neurological symptom burden, fatigue, and sleep quality. “Action-Only and NoEMA” was the best model that combined predictions of 1-sensor models trained on action-only features. “Action and Context and NoEMA” was the best model that combined predictions of 1-sensor models trained on action and context features. “Action-Only and AvgEMA” was the best model that combined predictions of 1-sensor models and the average EMA model trained on action-only features. “Action and Context and AvgEMA” was the best model that combined predictions of 1-sensor models and the average EMA model trained on action and context features. “Action-Only and PresurveyEMA” was the best model that combined predictions of 1-sensor models and the presurvey EMA model trained on action-only features. “Action and Context and PresurveyEMA” was the best model that combined predictions of 1-sensor models and the presurvey EMA model trained on action and context features.

^b^MS: multiple sclerosis.

^c^EMA: ecological momentary assessment.

#### Depressive Symptoms

For predicting the presence of depressive symptoms (vs the absence of depressive symptoms) every 2 weeks, the *baseline* model (simple majority classifier) had an accuracy of 59.5%. The model containing *all 6 sensors and no EMA* had an accuracy of 74.7% with action-only features (25.5% relative improvement over the baseline), and an accuracy of 72.2% with action and context features (21.3% relative improvement over the baseline; Table S2 in Multimedia Appendix 1). The model containing the *best combination of sensors and no EMA* had an accuracy of 74.7% with action-only features (25.5% relative improvement over the baseline; best combination: calls, heart rate, location, screen use, sleep, and steps), and an accuracy of 74.7% with action and context features (25.5% relative improvement over the baseline; best combination: calls, heart rate, location, screen use, and sleep; Table 2). The model containing the *best combination of sensors and average EMA* had an accuracy of 80.8% with action-only features (35.8% relative improvement over the baseline; best combination: heart rate, sleep, steps, and average EMA), and an accuracy of 81.3% with action and context features (36.6% relative improvement over the baseline; best combination: calls, heart rate, location, sleep, and average EMA). The model containing the *best combination of sensors and presurvey EMA* had an accuracy of 80.6% with action-only features (35.5% relative improvement over the baseline; best combination: heart rate, steps, and presurvey EMA) and an accuracy of 81.4% with action and context features (36.8% relative improvement over the baseline; best combination: heart rate, location, screen use, and presurvey EMA).

When comparing the model performance in a pairwise manner (Table 2), Action and Context and PresurveyEMA models had the highest bootstrapped average accuracy of 81.4% and the highest average *F*_1_-score of 0.77. This model significantly outperformed both NoEMA models: Action-Only and NoEMA (absolute increase of 6.7% in accuracy and 0.09 in *F*_1_-score) and Action and Context and NoEMA (absolute increase of 6.6% in accuracy and 0.1 in *F*_1_-score). Similarly, Action-Only and PresurveyEMA models significantly outperformed both NoEMA models: Action-Only and NoEMA (absolute increase of 6.0% in accuracy and 0.09 in *F*_1_-score) and Action and Context and NoEMA (absolute increase of 6.1% in accuracy and 0.09 in *F*_1_-score). Models with average EMA (Action-Only and AvgEMA, Action and Context and AvgEMA) also significantly outperformed both NoEMA models. However, there were no statistically significant differences between Action-Only and PresurveyEMA versus Action and Context and PresurveyEMA or between any of the PresurveyEMA models and the AvgEMA models.

Thus, for predicting the presence of depressive symptoms every 2 weeks, the *Action-Only and PresurveyEMA model* generated the best performance (accuracy=80.6%; *F*_1_-score=0.76) while requiring the least amount of sensor (eg, heart rate and steps) and EMA data (eg, presurvey EMA). Presurvey EMA was the last EMA response on the day before survey completion to assess patient-reported depressive symptoms.

#### Global MS Neurological Symptom Burden

For predicting high global MS neurological symptom burden (vs low burden) every 4 weeks, the baseline model had an accuracy of 51.1%. The model containing *all 6 sensors and no EMA* had an accuracy of 70.7% with action-only features (38.4% relative improvement over the baseline), and an accuracy of 72.0% with action and context features (40.9% relative improvement over the baseline; Table S2 in Multimedia Appendix 1). The model containing the *best combination of sensors and no EMA* had an accuracy of 77.3% with action-only features (51.3% relative improvement over the baseline; best combination: heart rate, location, sleep, and steps), and an accuracy of 73.8% with action and context features (44.4% relative improvement over the baseline; best combination: heart rate, location, and sleep; Table 2). The model containing the *best combination of sensors and average EMA* had an accuracy of 77.9% with action-only features (52.4% relative improvement over the baseline; best combination: heart rate, location, sleep, steps, and average EMA), and an accuracy of 79.7% with action and context features (56% relative improvement over the baseline; best combination: calls, heart rate, screen, sleep, and average EMA). The model containing the *best combination of sensors and presurvey EMA* had an accuracy of 78% with action-only features (52.6% relative improvement over the baseline; best combination: location, sleep, steps, and presurvey EMA) and an accuracy of 75.1% with action and context features (47.0% relative improvement over the baseline; best combination: heart rate, location, screen use, sleep, and presurvey EMA).

When comparing the model performance in a pairwise manner (Table 2), none was significantly better than the most parsimonious Action-Only and NoEMA model. Thus, for predicting high global MS symptom burden every 4 weeks, the *Action-Only* and *NoEMA* model generated the best performance (accuracy=77.3%; *F*_1_-score=0.77) while requiring the least amount of sensor data (ie, heart rate, location, sleep, and steps; trained on action-only features) and importantly no EMA data (ie, no active participant input).

#### Fatigue Impact

For predicting severe fatigue (vs mild fatigue) every 4 weeks, the baseline model had an accuracy of 50.9%. The model containing *all 6 sensors and no EMA* had an accuracy of 60.4% with action-only features (18.7% relative improvement over the baseline), and an accuracy of 69.7% with action and context features (36.9% relative improvement over the baseline; Table S2 in Multimedia Appendix 1). The model containing the *best combination of sensors and no EMA* had an accuracy of 67.6% with action-only features (32.8% relative improvement over the baseline; best combination: calls, heart rate, screen use, and steps), and 73.8% with action and context features (45% relative improvement over the baseline; best combination: heart rate, screen use, and steps; Table 2). The model containing the *best combination of sensors and average EMA* had an accuracy of 72.2% with action-only features (41.9% relative improvement over the baseline; best combination: heart rate, screen use, steps, and average EMA), and an accuracy of 76.1% with action and context features (49.5% relative improvement over the baseline; best combination: heart rate, screen use, sleep, steps, and average EMA). The model containing the *best combination of sensors and presurvey EMA* had an accuracy of 68.3% with action-only features (34.2% relative improvement over the baseline; best combination: heart rate, screen, steps, and presurvey EMA), and an accuracy of 77.1% with action and context features (51.5% relative improvement over the baseline; best combination: calls, heart rate, screen use, steps, and presurvey EMA).

When comparing the model performance in a pairwise manner (Table 2), none was significantly better than the Action and Context and NoEMA model. Thus, for predicting severe fatigue every 4 weeks, the *Action and Context* and *NoEMA* model generated the best performance (accuracy=73.8%; *F*_1_-score=0.74) while requiring the least amount of sensor data (ie, heart rate, screen use, and steps; trained on action and context features) and importantly no EMA data (ie, no active participant input).

#### Sleep Quality

For predicting poor sleep quality (vs better sleep quality) every 4 weeks, the baseline model had an accuracy of 56.2%. The model containing *all 6 sensors and no EMA* had an accuracy of 58.2% with action-only features (3.6% relative improvement over the baseline), and an accuracy of 68.7% with action and context features (22.2% relative improvement over the baseline; Table S2 in Multimedia Appendix 1). The model containing the best combination of sensors and no EMA had an accuracy of 72.0% with action-only features (28.1% relative improvement over the baseline; best combination: heart rate, location, sleep, and steps), and an accuracy 69.5% with action and context features (23.7% relative improvement over the baseline; best combination: calls, heart rate, sleep, and steps; Table 2). The model containing the *best combination of sensors and average EMA* had an accuracy of 74.4% with action-only features (32.4% relative improvement over the baseline; best combination: heart rate, location, screen, sleep, and average EMA), and an accuracy of 72.7% with action and context features (29.4% relative improvement over the baseline; best combination: heart rate, location, sleep, steps, and average EMA). The model containing the *best combination of sensors and presurvey EMA* had an accuracy of 72.0% with action-only features (28.1% relative improvement over the baseline; best combination: heart rate, location, sleep, and steps while presurvey EMA was not selected), and an accuracy of 74% with action and context features (31.7% relative improvement over the baseline; best combination: calls, heart rate, sleep, and presurvey EMA).

When comparing the model performance in a pairwise manner (Table 2), none was significantly better than the most parsimonious Action-Only and NoEMA model. Thus, for predicting poor sleep quality every 4 weeks, the *Action-Only and NoEMA* model generated the best performance (accuracy=72.0%; *F*_1_-score=0.7) while requiring the least amount of sensor data (ie, heart rate, location, sleep, and steps; trained on action-only features) and importantly no EMA data (ie, no active participant input).

## Discussion

### Principal Findings

For the primary goal of this study, which analyzed approximately 12,500 days of passively and continuously collected data from people with MS, we report the feasibility of a pragmatic and low-cost digital phenotyping approach that enables longitudinal tracking of common MS-related *patient-reported symptoms* in the patient’s own environment with minimal active patient engagement. Our approach harnesses passively collected sensor and behavior data from smartphones and fitness trackers and deploys ML models that achieve the highest prediction performance based on the most parsimonious data collection requirement. The key study finding is that, over 12 weeks (and 24 weeks in a subset), the best-performing models achieved potentially clinically actionable accuracy (as well as *F*_1_-score, which summarizes positive predictive value and sensitivity) for predicting the short-term presence of depressive symptoms (every 2 weeks), high global MS neurological symptom burden, severe fatigue, and poor sleep quality (every 4 weeks) in people with MS, all significantly outperforming the baseline models.

We consistently found that *heart rate*, *step count*, and *sleep* data outperformed other sensors, likely because they directly capture facets of physical activity, mobility, and rest patterns central to MS symptom fluctuations [6,7]. Unlike metrics, such as screen use or call logs, which are more likely to be confounded by external factors, heart rate and steps reflect exertion levels and functional status. Similarly, sleep data reveal potential disturbances in rest and circadian rhythm [40]. Consequently, these sensor streams may correlate more strongly with clinically meaningful outcomes in MS, aligning with previous findings that physical activity and sleep measures track disease-related disability and symptom burden more robustly than other behavioral metrics [8,11].

For a secondary study goal, we reported the marginal utility of behavioral features from the previous period (context features) in addition to behavioral features from the current period (action features) in helping the models contextualize an individual’s current behavior and in improving digital phenotyping of the most common MS symptoms. For each patient-reported symptom, we performed pairwise comparisons of the 6 best models combining sensor or sensor plus EMA (comprising action-only vs action and context features; Table 2) and operationally defined the “best” model as having the highest accuracy and *F*_1_-score while also requiring the least amount of sensor and EMA data. For predicting depressive symptoms, global MS neurological symptom burden, and sleep quality, the models containing action-only features were the “best” because the addition of context features did not improve the prediction of these patient-reported symptoms. In contrast, models containing action and context features improved the prediction of fatigue, a debilitating symptom for people with MS. Thus, behavioral features from longer periods that include context features (ie, the previous and current period) may have utility in the longitudinal symptom tracking of a smaller subset of common MS symptoms, such as fatigue. Methodologically, the addition of context features does not substantially increase complexity because context features derive from the same data streams and feature computation as action features, except for drawing data from the preceding period. Consequently, there is no additional data collection or substantial computational burden. Overall, context features may be valuable given the value of a longer temporal window for fatigue prediction while maintaining the same modeling pipeline.

For another secondary study goal, we reported the limited utility of incorporating minimal active patient input via EMA (ie, multiple-choice response to 2 brief survey questions) into ML models in improving digital phenotyping of the most common MS symptoms. For 3 of the 4 patient-reported symptoms (ie, global MS neurological symptom burden, fatigue, and sleep quality), the best models containing a combination of sensors plus average or presurvey EMA did not significantly outperform the best models containing a combination of sensors without EMA. Thus, passively collected sensor data without any active patient engagement were sufficient to predict the severity of these patient-reported symptoms. For fatigue, it is even more notable that 1 EMA question inquires tiredness (ie, “How tired do you feel?” on a 1-4 scale), which captures the transient and momentary feeling of tiredness as a *state*. In contrast, the patient-reported outcome based on MFIS-5 questionnaire measures the *impact* of fatigue on cognitive and physical function over the preceding 2 weeks. Thus, an individual may report feeling very tired at a given moment (ie, high EMA response score) but experience low impact of fatigue and still function effectively (ie, low MFIS-5 score). While the EMA question regarding momentary tiredness and MFIS-5 correlate, passively sensed behaviors are more likely to reflect the impact of fatigue on daily activities. This could explain why the EMA did not improve model performance in predicting the MFIS-5 score beyond passive sensor features. In contrast, for depression, the other EMA question (“How depressed do you feel?” on a 1-4 scale) aligns more closely with elements of the patient-reported outcome based on PHQ-9, focusing on emotional symptoms (rather than the impact of depressive symptoms), likely providing additional predictive value beyond passive sensor features. Overall, the EMA question of tiredness might be too simplistic or insensitive to capture the complexity and dynamics of fatigue impact on the physical, cognitive, and psychological function in people with MS [47,48]. For depressive symptoms, the best models containing a combination of sensors plus average or presurvey EMA *significantly outperformed* the best models containing a combination of sensors without EMA. While sensor data alone predicted depressive symptoms with reasonable accuracy (74.7%), the addition of presurvey EMA yielded an 8.8% absolute increase in accuracy. This result was unsurprising given that the other EMA question asked participants to rate the level of depressed feeling. Notably, the best models containing presurvey EMA were comparable to those containing average EMA, while presurvey EMA (ie, the last EMA on the day before the patient-reported symptom survey) required substantially less active engagement by participants than average EMA (ie, 3 times daily). Overall, minimally active participant engagement may have some utility in the longitudinal symptom tracking of certain MS symptoms, such as depressive symptoms.

Broadly, several aspects of our study differentiated from previous works, all with the goal of bringing digital phenotyping closer to clinical practice for people with MS. First, to demonstrate a basic feature of real-world applicability, our pragmatic study design leveraged each participant’s *own* digital device (eg, smartphone) to mitigate missing sensor data capture. In contrast, the earliest studies required a study-specific smartphone separate from participant’s own smartphone and increased participant’s burden [49]. Second, our approach passively harnessed data from a combination of multiple sensors in both smartphones and fitness trackers. Previous studies predicting MS outcomes based on passively sensed behavior largely relied on either a smartphone or fitness tracker (but not both) or a single sensor type [6,7,11,13]. Third, our ML pipeline prioritized the *most parsimonious predictive models* containing the least amount of sensor and EMA data (ie, minimal or no active participant engagement) while still achieving clinically actionable accuracy and other prediction metrics. By comparison, most previous digital phenotyping efforts in MS prioritized performance without considering the amount of sensor data required for prediction and indeed often required active participant engagement, which would lead to lower adherence than passive sensing [9,49-56]. For instance, the study by Gashi et al [9] required participants to perform motor performance tests to classify fatigue levels in addition to passively sensed behavioral data. Finally, our study outcomes as measured by validated survey instruments included a spectrum of common clinically relevant *patient-reported symptoms* that collectively reduce the quality of life in people with MS. In contrast, standard clinical trial end points, such as clinician-rated disability or functional testing scores [6,8,9,13,14,57-65], as well as a single clinical outcome (at a time) [54,66-69] in previous studies insufficiently captured the full real-world patient experience.

This study also built on one of our own previous studies, which used passively sensed behavior changes during a state-mandated stay-at-home period (as compared to the prepandemic baseline) to predict depressive symptom, high global MS symptom burden, severe fatigue, and poor sleep quality in people with MS in a unique natural experiment in the setting of a global pandemic [19]. Specifically, we predicted the average value of patient-reported outcomes for each patient only once during a period (ie, the local COVID-19 stay-at-home period), whereas this study made repeated clinical predictions (over consecutive 2- or 4-week periods) during a 12- or 24-week study duration to emulate long-term symptom tracking in the real world. As methodological novelties, this study further investigated the added utility of context behavioral features (from the previous periods) and 2 types of EMAs in improving digital phenotyping in MS.

Our digital phenotyping approach, with *minimal or no active patient input* that reaches potentially clinically actionable prediction performance, warrants additional investigations of its future clinical role in continuous tracking of *patient-reported symptoms* and in assisting comprehensive MS care in the real-world setting. Timely management of these common patient-reported symptoms could reduce delays in symptom management and greatly improve the quality of life for people with MS. Of clinical relevance, patient-reported symptoms assessed in this study are based on well-validated survey instruments that correlate with and complement clinician-rated outcomes. Practically, one can envision deploying continuous digital phenotyping to enable not only patient self-monitoring between routine clinic appointments but also crucial clinical triage for timely interventions (eg, medication initiation and counseling). Such an approach may even be potentially useful in settings of limited health care access and resources, though such clinical application would require dedicated testing.

While this proof-of-concept study demonstrates the potential clinical applications of digital phenotyping, several challenges will require solutions for eventual successful real-world implementation. First, patient adherence (eg, wearing or carrying the devices and keeping devices charged) is a key prerequisite, as continuous passive sensing relies on consistent use of a charged device. While participants in this study demonstrated high levels of engagement and adherence, successful monitoring requires frequent supervision of real-time adherence by the research staff. An effective real-world implementation will require pragmatic techniques that maintain patient adherence without increasing patient or clinician burden. For example, future pragmatic strategies to improve adherence may include artificial intelligence coupled with automated reminders and user-friendly device designs. Second, easily interpretable findings from the digital phenotyping data are crucial to translate into clinical action and for incorporation into clinical practice. Using clinically meaningful binary thresholds of common symptom severity is one example of clinical interpretability. Third, eventual clinical implementation of digital phenotyping will require a rigorous regulatory approval process, acceptance by health care systems based on cost-effectiveness as added value, and technical integration with existing electronic health records. Finally, the digital health and clinical community will need to carefully safeguard data privacy and potential biases in ML models. In particular, we will ensure secure data storage, transparent model interpretability, and equitable algorithm performance across broad patient populations.

### Limitations

Our study has at least 2 limitations. First, the study participant size, while larger than most previous digital phenotyping studies in MS, was still relatively modest. We made predictions for >700 samples for depressive symptoms (in 2-week periods) and >300 samples for global MS neurological symptom burden, fatigue, and sleep quality (in 4-week periods) across 104 participants with MS. Notably, our well-characterized cohort also contrasts with larger studies where the diagnosis and patient-reported outcomes could not be independently verified [10,55]. Crucially, we mitigated model overfitting using leave-5-participants-out-cross-validation such that the participants used for training and testing were different in each fold. The consistently robust model performance across all 5 folds and for all 4 common reported symptoms by participants was reassuring. Second, we recruited study participants from a single clinic-based cohort, representative of its local patient population. While MS is a disease predominantly affecting women of European descent, the high proportion of White and female participants in the study limits the generalizability of the specific findings, though the potential clinical implications are still valid as a proof-of-concept study. Future validation in external cohorts with more racially and ethnically diverse patient populations would improve the generalizability of the approach.

### Conclusions

In summary, our digital phenotyping approach using passively sensed data from patients’ own smartphones and wearable fitness trackers could aid them with real-world, continuous, self-monitoring of common symptoms in their native environment. It may also assist clinicians with better triage of patient needs for timely intervention in MS and potentially other chronic neurological disorders.

## Appendix

Multimedia Appendix 1 Additional methods (details of feature extraction and engineering and handling missing data) and additional results for different sensor combinations and configurations.

## Abbreviations

EMA: ecological momentary assessment
MFIS-5: Modified Fatigue Impact Scale-5
ML: machine learning
MS: multiple sclerosis
MSRS-R: Multiple Sclerosis Rating Scale-Revised
PHQ-9: Patient Health Questionnaire-9
PSQI: Pittsburgh Sleep Quality Index
RBF: radial basis function
SVM: support vector machine
